# Prognostic biomarker DARS2 correlated with immune infiltrates in bladder tumor

**DOI:** 10.3389/fimmu.2023.1301945

**Published:** 2024-01-17

**Authors:** Hailang Yang, Li Ma, Wen Deng, Bin Fu, Jianqiang Nie, Xiaoqiang Liu

**Affiliations:** ^1^Department of Urology, The First Affiliated Hospital, Jiangxi Medical College, Nanchang University, Nanchang, China; ^2^Department of Pathology, The First Affiliated Hospital, Jiangxi Medical College, Nanchang University, Nanchang, China; ^3^Jiangxi Institute of Urology, Nanchang, China

**Keywords:** DARS2, bladder cancer, prognosis, immune infiltration, PD-L1

## Abstract

**Background:**

DARS2 is a pivotal member of the Aminoacyl-tRNA synthetases family that is critical for regulating protein translation. However, the biological role of DARS2 in bladder cancer remains elusive.

**Methods:**

We analyzed the correlation between DARS2 expression and prognosis, tumor stage, and immune infiltration in bladder cancer using The Cancer Genome Atlas (TCGA) database. We validated findings in clinical samples from The First Affiliated Hospital of Nanchang University and explored the biological functions of DARS2 using cell and animal models.

**Results:**

We found DARS2 to be upregulated in bladder cancer, associated with tumor progression and poor prognosis. Immune infiltration analysis suggested that DARS2 may facilitate immune evasion by modulating PD-L1. Cell and animal experiments validated that DARS2 knockdown and overexpress can inhibit or increase cancer cell proliferation, metastasis, tumorigenesis, immune escape, and PD-L1 levels.

**Conclusions:**

Our study reveals DARS2 as a potential prognostic biomarker and immunotherapy target in BLCA.

## 1 Introduction

Bladder cancer (BLCA) is the most common malignancy of the urinary tract and one of the most prevalent cancers worldwide (1). Globally, it is the ninth most prevalent cancer, and among males, it ranks sixth most common (2). Approximately 70-80% of patients are diagnosed with non-muscle-invasive BLCA at the initial diagnosis. However, up to 20% of patients progress to advanced, high-grade muscle-invasive BLCA, with a 5-year survival rate of less than 50% (3). Despite significant advancements in the treatment of BLCA, including targeted therapies and immunotherapy, the survival rates for BLCA have not shown significant improvement over the past three decades (4, 5). Therefore, investigating the underlying mechanisms of BLCA development and identifying novel therapeutic targets is of paramount importance for improving patient prognosis.

aminoacyl-tRNA synthetases (ARS) are critical enzymes that catalyze the synthesis of proteins by transferring amino acids onto their corresponding homologous transfer RNAs (tRNAs) (6–8). ARS constitutes an evolutionarily conserved and essential enzyme family responsible for catalyzing the linkage of tRNA with its cognate amino acid, facilitating translation (9).ARS was once considered a family of ‘housekeeping’ enzymes; however, it is now known that they play diverse roles, including involvement in transcription, translation, splicing, inflammation, angiogenesis, and apoptosis. ARS also serves as regulators and signaling molecules in various immune diseases, infectious diseases, and tumor immunity (9–11).

The gene encoding mitochondrial Aspartyl-tRNA synthetase 2 (DARS2), DARS2 encodes mitochondrial ARS, which specifically catalyzes the aminoacylation of aspartyl-tRNA. Mutations in this gene are associated with leukoencephalopathy with brainstem and spinal cord involvement and lactate elevation, a white matter brain disorder characterized by brainstem and spinal cord involvement and elevated lactate levels (12, 13). DARS2 is a significant member of the ARS family and is implicated in tumorigenesis (14, 15). Sukru’s group discovered that the deficiency of DARS2 leads to the activation of various stress responses in a tissue-specific manner. Depletion of DARS2 in the heart and skeletal muscles results in a severe disruption of mitochondrial protein synthesis (14, 15). It has been discovered that HBV inhibits NFAT5 via the miR-30e-5p/mitogen-activated protein kinase signaling pathway upstream of NFAT5. Furthermore, HBV enhances hepatocellular carcinoma tumor development by suppressing NFAT5 through downstream target genes, including DARS2 (16). It reported an upregulation of DARS2 expression in lung adenocarcinoma and highlighted its role in regulating the proliferation, invasion, and apoptosis of lung adenocarcinoma cells. Moreover, DARS2 overexpression was associated with poor prognosis in lung adenocarcinoma (17). However, the biological role of DARS2 in BLCA has not yet been completely researched.

In this study, we investigated the expression levels of DARS2 in BLCA and corresponding adjacent tissues, analyzing the infiltration of immune cells. We further explored the association between DARS2 expression and overall survival. Additionally, we delved into the impact of DARS2 interference on tumor cell proliferation, invasion, migration, and PD-L1 expression. To gain a more comprehensive understanding of its role in tumor immune regulation, we conducted co-culture experiments with DARS2 and Jurkat cells, with further validation through animal experiments. Collectively, all these research findings affirm the potential of DARS2 as a novel prognostic marker guiding BLCA treatment.

## 2 Methods

### 2.1 Data source

mRNA expression data and clinical information for BLCA were downloaded from The Cancer Genome Atlas (TCGA) (https://portal.gdc.cancer.gov), while pan-cancer data for DARS2 were obtained from TIMER2 (http://timer.cistrome.org/) (18).To further validate the protein expression of DARS2 in BLCA, we collected surgical specimens of BLCA and normal bladder tissues from six patients during surgery. Additionally, we selected pathological specimens from 37 BLCA samples and 10 normal bladder tissue samples at the First Affiliated Hospital of Nanchang University for immunohistochemistry (IHC) and collected clinical information from these patients. All patient specimens underwent histological diagnosis by two pathologists. This study was approved by the Ethics Committee of the First Affiliated Hospital of Nanchang University, Ethics license number (2022):CDYFYYLK (11–031); CDYFY- IACUC-202308QR018.

### 2.2 Survival analysis

Patients were stratified into high-expression and low-expression groups based on DARS2 expression levels in the TCGA dataset and IHC expression in specimens collected from the First Affiliated Hospital of Nanchang University. OS (Overall Survival) analysis was performed using the ‘survival’ and ‘survminer’ R packages to analyze and visualize survival information for both the TCGA dataset and patients collected from the First Affiliated Hospital of Nanchang University.

### 2.3 Immunoinfiltration and gene expression correlation analysis

TIMER2 is a comprehensive resource for studying molecular features of tumor-immune interactions across various cancer types (https://cistrome.shinyapps.io/timer/) (18). We utilized TIMER2 to analyze the correlation between DARS2 expression and the infiltration of seven immune cell types, including CD4^+^T cells, CD8^+^T cells, B cells, NK (Natural Killer) cells, macrophages, MDSCs (Myeloid-Derived Suppressor Cells), CAFs (Cancer-Associated Fibroblasts), macrophages, and Treg (Regulatory T) cells.

We also analyzed the genes of three immune checkpoints, including PD1 (Programmed Cell Death 1, also known as PDCD1), PD-L1 (Programmed Cell Death 1 Ligand 1, also known as CD274), and CTLA4 (Cytotoxic T-Lymphocyte-Associated Protein 4), using the Genetic correlation module on TIMER2 (adjusted for tumor purity) with statistical methods based on Spearman’s rank correlation coefficient. Furthermore, we conducted preliminary validation of DARS2 and PD-L1 expression using five pairs of BLCA tissues.

### 2.4 Gene set enrichment analysis

Performing Gene Set Enrichment Analysis (GSEA) using gene sets from the MSigDB collection between the high and low DARS2 expression groups (19). Identifying potential signaling pathways regulated by DARS2 using the ‘clusterProfiler’ package.

### 2.5 Cell culture and transfection

The human urothelial carcinoma cell lines T24, J82, EJ, and the normal urothelial cell line SV-HUC were obtained from the Cell Bank of the Shanghai Institute of Cell Biology, Chinese Academy of Sciences, China. T24, J82, EJ, and SV-HUC cells were cultured in DMEM, RPMI-1640, and F12K media, respectively, supplemented with 10% fetal bovine serum (FBS, Hyclone) and 100 U/mL penicillin/streptomycin, and were grown at 37°C with 5% CO_2_. T24 and EJ cells were seeded in six-well plates at a density of 2x10^5^ cells per well.

### 2.6 Primers and SiRNA knockdown fragments

DARS2:

Forward Primer: CGAGATGAAGGTTCAAGACCAGA

Reverse Primer: GCCAGGAATACTGGAGCAAACC

β-Actin:

Forward Primer: TCTTCCAGCCTTCCTTCCT

Reverse Primer: AGCACTGTGTTGGCGTACAG

DARS2 SiRNA Interference Fragments:

si-1: GCGTAGTTTCCAAATGCAGTA

si-2: GCCACCTATGGAACTGATAAA

si-3: GCCAACACTATGACTTGGTTT

Lentiviral Interference Fragment (shRNA):

shRNA: GCGTAGTTTCCAAATGCAGTA

Lentiviral Vector: pLV3-U6-MCS-shRNA-EF1a-CopGFP-Puro

Overexpression DARS2:

Forward Primer: TGCTCGCCTTCCTCTTTCAG

Reverse Primer: AGGGAGGCTAAGCGAGGTTT

### 2.7 Jurkat cell co-culture system

Tumor cells were seeded onto a 12-well plate and allowed to adhere. Following attachment, Jurkat cells, pre-cultured for 24 hours with 1ug/mL PHA and 50ng/mL PMA, were introduced into the upper chamber of a migration apparatus. The semi-permeable membrane of the migration chamber, featuring a pore size of 0.4um, adeptly prevented reciprocal cell transmigration between the upper and lower compartments. Maintaining a proportion of 1:8 for tumor cells to Jurkat cells, a 24-hour co-cultivation ensued, with subsequent collection of culture medium for IL-2(Human IL-2 ELISA Kit EK102, MULTISCIENCES) level assessment through ELISA. Concurrently, CCK-8 reagent was employed to evaluate the cell viability of tumor cells in both the control group (without Jurkat cells group) and the co-culture group. Finally, the co-cultured tumor cells were subjected to crystal violet staining to portray any remaining cells visually.

### 2.8 Other reagents

DARS2 antibody: Sourced from Wuhan Boster Biotechnology Co., Ltd., Catalog Number A06034-1.GAPDH antibody: Obtained from Wuhan Sanying Biotechnology Co., Ltd., Catalog Number 60004-1-Ig.Ki67 antibody:Obtain from Wuhan Servicebio, GB151142-100. Anti-mouse HRP-conjugated secondary antibody: Supplied by Wuhan Sanying Biotechnology Co., Ltd., Catalog Number KFA025.Anti-rabbit HRP-conjugated secondary antibody: Provided by Wuhan Sanying Biotechnology Co., Ltd., Catalog Number KFA005.PD-L1 antibody: Procured from Wuhan Sanying Biotechnology Co., Ltd., Catalog Number 28076-1-AP.Lipo 2000 transfection reagent: Purchased from Sigma-Aldrich, Massachusetts, USA.PEI transfection reagent: Obtained from Wuhan Sanying Biotechnology Co., Ltd. DAB staining solution: Sourced from Background Soledad Technology Co., Ltd.

### 2.9 Ethical approval

This study was approved by the Ethics Committee of the First Affiliated Hospital of Nanchang University, Ethics license number (2022):CDYFYYLK (11–031); CDYFY- IACUC-202308QR018.

## 3 Results

### 3.1 Upregulation of DARS2 expression in BLCA

**Figure 1:**
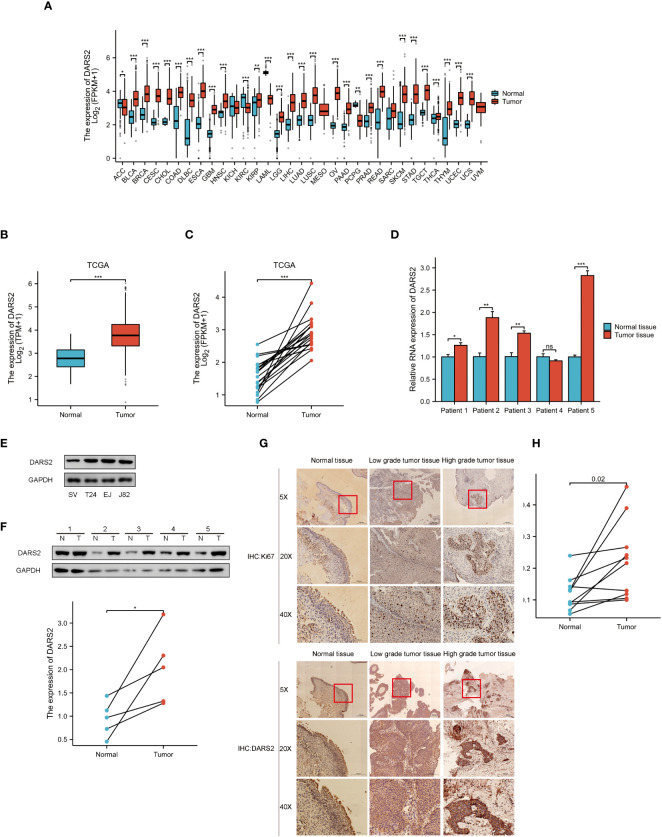
DARS2 Expression in BLCA. **(A)** DARS2 Pan-Cancer Analysis. **(B)** TCGA Analysis of DARS2 Expression in BLCA. **(C)** The expression of DARS2 in paired bladder cancer in TCGA. **(D)** qPCR analysis of DARS2 expression in 5 pairs of bladder cancer tissues. **(E)** Expression of DARS2 in bladder cancer cells and normal urothelial cells by western blot. **(F)** Western blot analysis of DARS2 expression in 5 pairs of bladder cancer tissues. **(G)** Immunohistochemistry of DARS2 and Ki67 in bladder cancer tissues. **(H)** Immunohistochemical analysis of DARS2 in 10 pairs of bladder cancer tissues. *P<0.05, **P<0.01, ***P<0.001. ns, no statistical difference.

We analyzed TCGA data to investigate the expression of DARS2 in tumor and normal tissues. Our findings revealed that DARS2 expression was significantly higher than that in normal tissues across 25 types of tumors, such as BLCA, BRCA, CESC,etc (**Figure 1A**). Furthermore, our analysis of TCGA data demonstrated a significant upregulation of DARS2 expression in BLCA (P<0.001, **Figure 1B**). Additionally, analysis of paired mRNA expression data from TCGA showed a significant increase in DARS2 expression in tumor tissues (P<0.001, **Figure 1C**).

Subsequently, qPCR was performed to validate the mRNA expression levels of DARS2 in BLCA using paired samples from five patients. The results showed an increase in DARS2 mRNA expression in four pairs of cancer tissues compared to adjacent normal tissues (**Figure 1D**). Protein immunoblotting results further demonstrated elevated expression of DARS2 in both cancer cell lines and tumor tissues (**Figures 1E, F**). We employed Ki67 to assess the malignancy level of ten pairs of bladder cancer tissues, because a higher proportion of Ki67-positive cells correlates with increased malignancy. Immunohistochemical (IHC) analysis of the ten pairs of bladder cancer tissues indicated a heightened expression of DARS2 in bladder cancer compared to normal bladder mucosal epithelial tissues (P<0.05, **Figures 1G, H**). Additionally, we observed that high-grade bladder cancer exhibited elevated levels of DARS2 expression compared to low-grade bladder cancer. This trend was consistent with the Ki67 staining results, where high-grade bladder cancer showed a higher proportion of Ki67-positive cells compared to the low-grade counterpart.

Subsequently, patients were grouped based on clinical characteristics to determine the correlation between DARS2 expression levels and clinical features. TCGA analysis results revealed significant differences in higher DARS2 expression across histological grade (P < 0.001), pathological stage (P < 0.05), T stage (P < 0.05), and M stage (P < 0.01) (**Supplementary Table 1**). Furthermore, to further confirm the relationship between clinical features of tumors and DARS2 protein expression, we conducted clinical baseline data analysis based on the results of IHC analysis in 37 patient samples. The patients were divided into high and low DARS2 expression groups based on the median DARS2 expression. The analysis showed significant differences in higher DARS2 expression concerning N stage (P < 0.05), M stage (P < 0.05), and histological grade (P < 0.05) (**Supplementary Table 2**). Notably, the results for M stage and histological grade from IHC analysis were consistent with the TCGA results.

In summary, these findings suggest that DARS2 expression is upregulated in BLCA at both the transcriptional and translational levels.

### 3.2 DARS2 as an independent prognostic factor in BLCA

**Figure 2:**
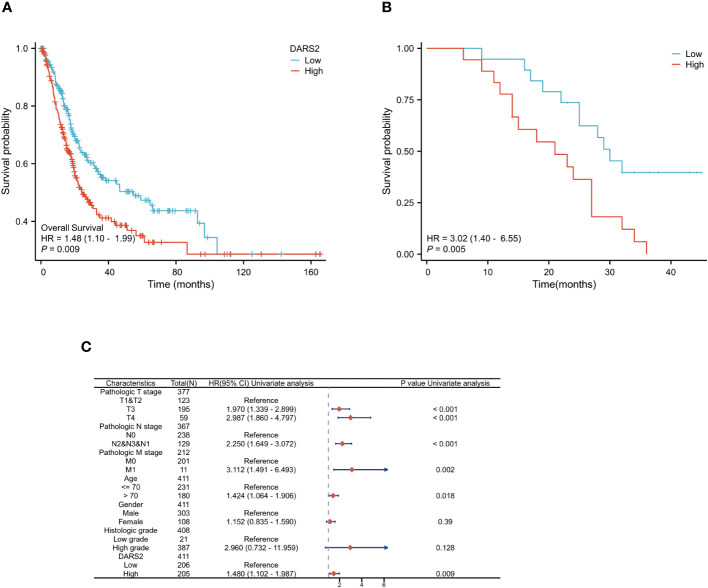
DARS2 as an Independent Prognostic Factor in BLCA. **(A)** Kaplan-Meier analysis of OS in the TCGA BLCA. **(B)** Kaplan-Meier analysis of OS in the 37 cases of BLCA immunohistochemistry. **(C) **DARS2 expression distribution and survival status.

To determine the prognostic value of DARS2 in BLCA, we divided patients in the TCGA dataset into DARS2 low-expression and DARS2 high-expression groups based on the median DARS2 expression for survival analysis. High DARS2 expression was associated with poorer overall survival (OS) in BLCA (HR = 1.48, P < 0.01) (**Figure 2A**).

To further validate the prognostic role of DARS2, we analyzed follow-up data from collected patients. Based on IHC analysis results, patients were categorized into high-expression and low-expression groups. The analysis revealed that patients in the high-expression group had worse OS (HR = 3.02, P < 0.01) (**Figure 2B**).

Next, we performed univariate and multivariate Cox regression analyses on clinical data from TCGA to determine the correlation between overall survival in BLCA and multiple factors. Univariate analysis showed that six clinical features, including T3 stage (HR = 1.970, P < 0.001), T4 stage (HR = 2.987, P < 0.001), N stage (HR = 2.250, P < 0.001), M stage (HR = 3.112, P < 0.005), age >70 (HR = 1.424, P < 0.05), and DARS2 expression (HR = 1.480, P < 0.005), were significantly associated with patient overall survival (**Supplementary Table 3**; **Figure 2C**). Multivariate analysis data revealed that DARS2 expression (HR = 1.953, P = 0.022) is an independent prognostic factor (**Supplementary Table 3**).

### 3.3 Identification and enrichment analysis of differentially expressed genes

**Figure 3:**
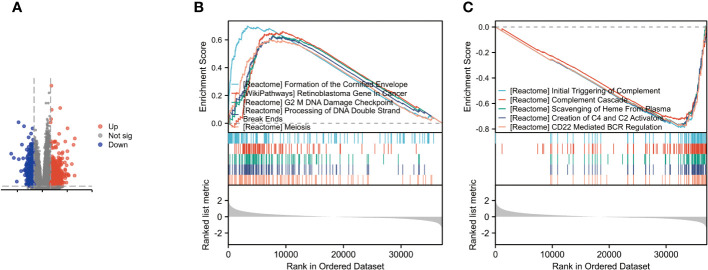
Identification and Enrichment Analysis of Differentially Expressed Genes. **(A)** Volcano plot for differentially expressed genes between high and low expression of DARS2 in BLCA patients. **(B) **GSEA revealed the top five are positively correlated. **(C)** GSEA revealed the top five are poorly correlated.

Through gene differential analysis, a total of 466 genes were identified as differentially expressed genes (DEGs) between the high DARS2 group and the low DARS2 group (**Figure 3A**). To identify signaling pathways regulated by abnormal DARS2 expression, we compared the DARS2 high-expression and low-expression groups using a signature gene set based on the TCGA dataset.

The analysis results indicated the top five upregulated pathways as follows: Formation of the cornified envelope; WP retinoblastoma gene in cancer; G2 mDNA damage checkpoint; Processing of DNA double-strand break ends; Meiosis (**Figure 3B**)

Conversely, the top five downregulated pathways were identified as Initial triggering of complement; Complement cascade; Scavenging of heme from plasma; Creation of C4 and C2 activators; and CD22 mediated by regulation (**Figure 3C**)

These findings provide insights into the potential pathways and processes influenced by DARS2 dysregulation in BLCA, offering valuable information for further mechanistic investigations and therapeutic targeting.

### 3.4 DARS2 regulates bladder cancer cell proliferation, migration, and invasion

**Figure 4:**
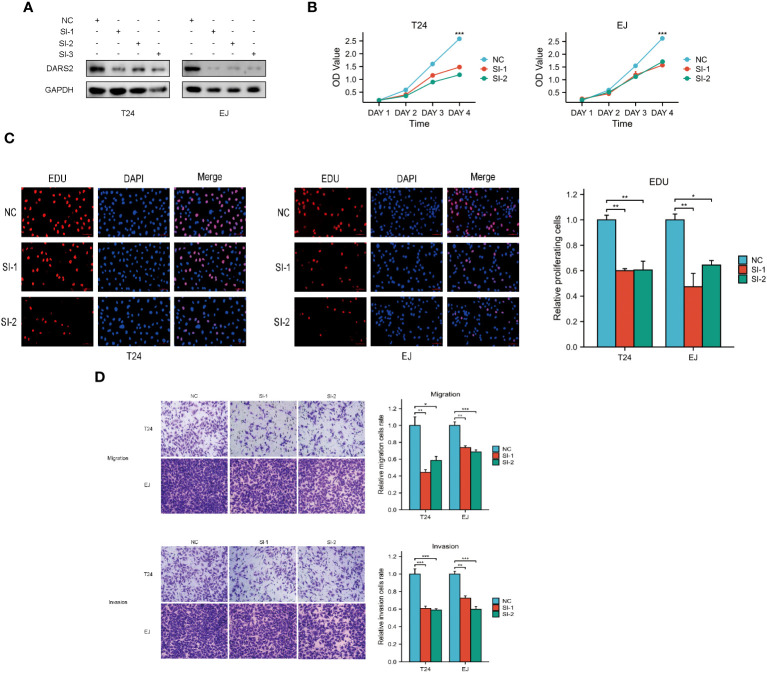
DARS2 Regulates Bladder Cancer Cell Proliferation, Migration, and Invasion. **(A)** DARS2 knockdown in T24 and EJ cells. **(B, C)** The impact of DARS2 on proliferation in T24 and EJ cells via CCK8, EDU. **(D)** The impact of DARS2 on migration and invasion in T24 and EJ cells. *P<0.05, **P<0.01, ***P<0.001.

The above results indicate that DARS2 is upregulated in BLCA, suggesting its crucial role in BLCA tumorigenesis. Therefore, we used siRNA to knock down DARS2 expression (**Figure 4A**) to observe its biological effects on T24 and EJ cells. We chose Si-1 and Si-2 for cell experiments. We assessed the impact of DARS2 on cell proliferation using CCK8 and EDU assays. Our results showed that knocking down DARS2 inhibited cell proliferation (**Figures 4B, C**). Furthermore, we found that migration and invasion abilities were reduced in T24 and EJ cells with reduced DARS2 expression (**Figure 4D**).

In addition, we conducted an overexpression experiment of DARS2 in bladder cancer cells and assessed relevant indicators. Our research revealed that the overexpression of DARS2 significantly promotes cell proliferation, invasion, and migration, in stark contrast to the experimental results of DARS2 knockdown (**Supplementary Figures 1A-D**).

In summary, these results indicate that DARS2 can affect cell proliferation, migration, and invasion.

### 3.5 DARS2 expression correlates with immune infiltration and PD-L1 expression

Our analysis using TIMER2 showed that DARS2 expression was negatively correlated with immune-active cells, including CD4^+^ T cells (R= -0.251, P < 0.001) and NK cells (R= -0.067, P < 0.001). Conversely, DARS2 expression was positively correlated with immunosuppressive cells, such as MDSCs (R= 0.372, P < 0.001) and macrophages (R= 0.196, P < 0.01). Interestingly, DARS2 expression was positively correlated with CD8^+^T cells (R= 0.203, P < 0.001) (**Supplementary Figure 2A**).

It is known that blocking immune checkpoint receptors such as PD-1/PD-L1 and CTLA-4 can alleviate CD8^+^T cell exhaustion and reactivate immune cell cytotoxicity to eliminate antigen-expressing tumor cells (20). Therefore, we analyzed the correlation between DARS2 expression and the expression of PD-1, PD-L1 (CD274), and CTLA-4 using TIMER2. We found that DARS2 expression was positively correlated with PD-L1 expression (R= 0.202, P < 0.001), but showed no significant correlation with PD-1 and CTLA-4 (**Supplementary Figure 2B**).

**Figure 5:**
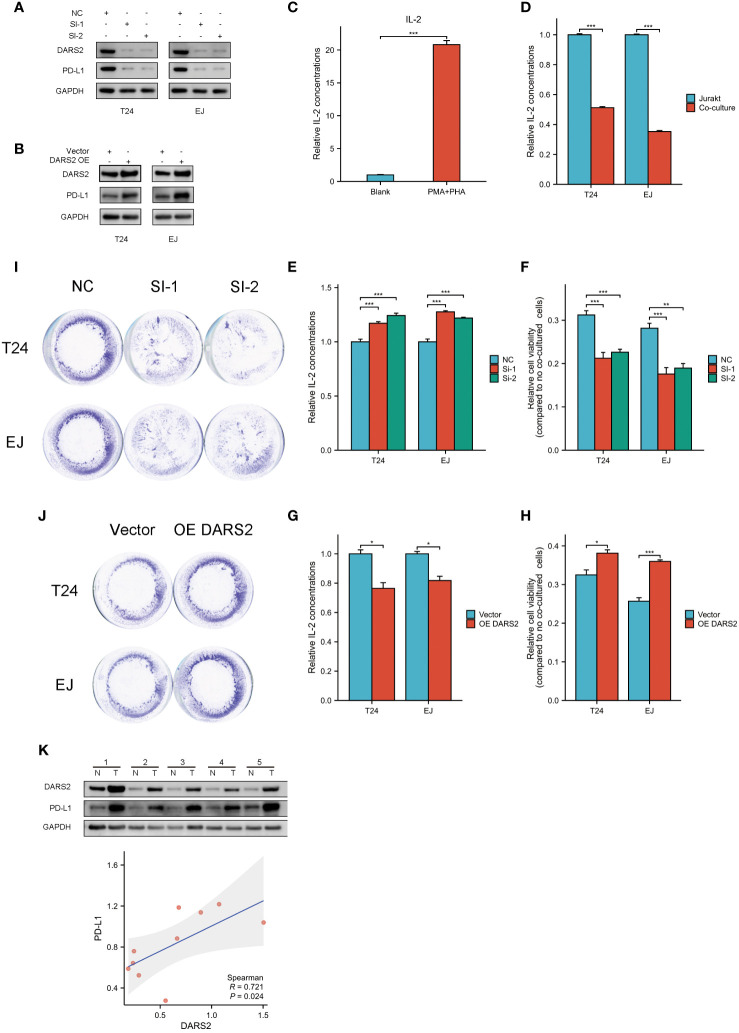
Bladder cancer cells and Jurkat cells co-cultured. **(A, B)** PD-L1 changes after interference and overexpression of DARS2. **(C)** Relative concentration of IL-2 in culture medium after activation of jurkat cells by PMA and PHA. **(D)** Relative concentration of IL-2 in the culture medium after co-culture of activated Jurkat cells and untreated bladder cancer cells. **(E, G)** Relative concentration of IL-2 in the co-culture system after knocking down and overexpressing DRAS2.**(F, H)** Viability of residual surviving tumor cells in co-culture system after knockdown and overexpression of DRAS2 **(I, J)** Crystal violet staining of remaining surviving tumor cells in the co-culture system after knocking down and overexpressing DRAS2. **(K)** Expression of DARS2 and PD-L1 in bladder cancer cells and normal urothelial cells by western blot and analysis of the correlation between shigeDARS2 and PD-L1. *P<0.05, **P<0.01, ***P<0.001.

To validate the expression patterns of DARS2 and PD-L1, we conducted protein immunoblotting on five pairs of clinical samples derived from patients. The results revealed elevated expression of both DARS2 and PD-L1 in cancer tissues compared to adjacent non-cancerous tissues across all five sample sets. Furthermore, correlation analysis of the results from 10 samples demonstrated a positive association between the expression levels of DARS2 and PD-L1 (**Figure 5K**).

Subsequently, we performed knockdown and overexpression experiments targeting DARS2. Following interference or overexpression of DARS2, we observed a corresponding downregulation or upregulation of PD-L1 expression, respectively(**Figures 5A, B**). In response to these findings, we designed co-culture experiments to investigate the impact of cellular immunity of DARS2 on PD-L1 expression.

We established a co-culture model. We induced Jurkat cell activation with 50ng/mL PMA and 10ug/mL PHA for 24 hours, resulting in increased IL-2 levels post-activation (**Figure 5C**). Subsequently, using a 12-well plate, we co-cultured cells, either solely Jurkat cells or Jurkat cells in a 1:5 ratio with tumor cells, for 24 hours. Previous research has indicated that high PD-L1 expression can inhibit the function of Jurkat cells, leading to a reduction in IL-2 expression (21).Consistent with the mentioned study, we observed a decrease in IL-2 levels in the co-culture medium containing tumor cells (**Figure 5D**).

We then conducted co-cultures after knockdown and overexpression of DARS2, maintaining conditions consistent with the explored co-culture settings. Notably, the knockdown of DARS2 resulted in an increase in IL-2 levels in the co-culture medium, while overexpression led to a decrease in IL-2 levels (**Figures 5E, G**).

Finally, we investigated the activity of residual tumor cells after co-culture. In the co-culture setting, we observed lower activity in tumor cells of the DARS2 knockdown group compared to the control group. Conversely, overexpression of DARS2 resulted in heightened activity of tumor cells in the co-culture (**Figures 5F, H**). Crystal violet staining of residual live cells post-co-culture aligned with the observed cell viability patterns (**Figures 5I, J**).

This indicates that in the immune microenvironment, elevated expression of DARS2 can lead to a reduction in the cytotoxicity of Jurkat cells against tumor cells. Conversely, lower expression of DARS2 results in increased susceptibility of tumor cells to Jurkat cell-mediated cytotoxicity. PD-L1 plays a crucial role in the cytotoxic process of Jurkat cells against tumor cells. Moreover, DARS2 has the ability to influence the expression of PD-L1.

Therefore, DARS2 expression plays a significant role in immune infiltration and may serve as a potential biomarker for immune therapy response in BLCA patients.

### 3.6 Knockdown of DARS2 inhibits tumor formation in nude mice

**Figure 6:**
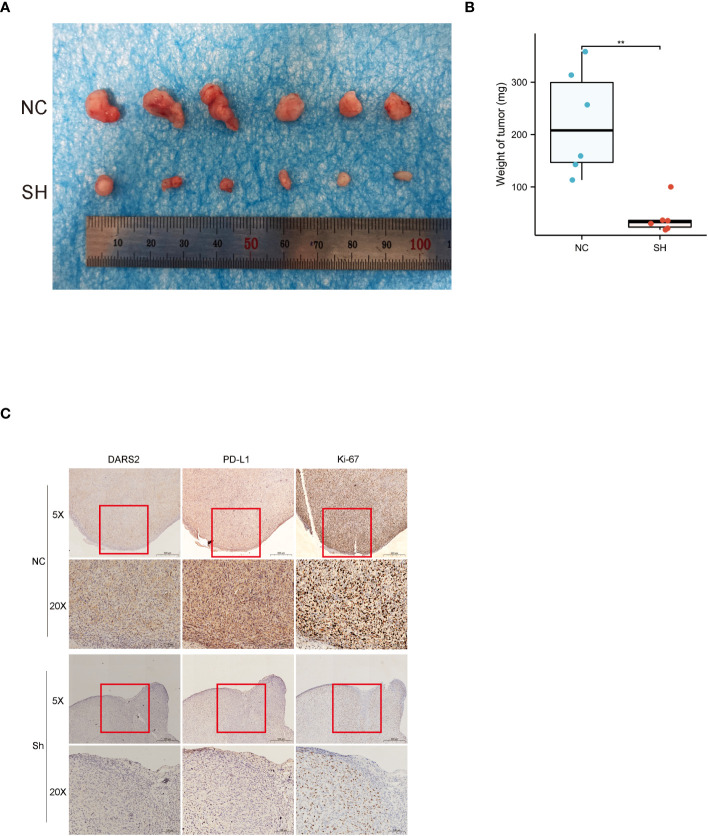
**(A, B)** subcutaneous tumor formation in nude mice. **(C)** Immunohistochemical staining of DARS2, PD-L1 and Ki67 in subcutaneous tumor samples from nude mice. *P<0.05, **P<0.01, ***P<0.001.

In order to further study the impact of DARS2 on bladder cancer, we used T24 cells to conduct tumorigenesis experiments in nude mice.we found that T24 cells with DARS2 knockdown formed smaller subcutaneous tumors in nude mice compared to the NC group in animal experiments (**Figures 6A, B**). IHC images of tumor tissues showed a decrease in both DARS2, cell proliferation markers Ki67 and the immune marker PD -L1 after DARS2 knockdown (**Figure 6C**). The above experiments show that DARS2 can affect the proliferation and tumor formation of bladder cancer.

## 4 Discussion

BLCA remains one of the most common malignancies of the urinary tract and one of the most prevalent cancers globally (1, 2). Approximately 70-80% of patients are diagnosed with non-muscle-invasive BLCA initially, but up to 20% progress to high-grade, high-stage muscle-invasive BLCA, with a 5-year survival rate of less than 50% (3, 22). Despite the significant advances in BLCA treatment, such as targeted therapies and immunotherapies, the survival rates for BLCA have not significantly improved (1, 23). Therefore, it is crucial to explore novel biomarkers for BLCA.

DARS2 is responsible for producing the enzyme that ensures the correct translation of the genetic code by attaching amino acids to their corresponding tRNA molecules in the mitochondria (9, 11, 24). Liu and colleagues discovered that DARS2 can serve as a prognostic marker for non-adenocarcinomas and promote the proliferation, invasion, and migration of lung adenocarcinoma cells while inhibiting cell apoptosis (17, 25, 26). It suggests that DARS2 may be proposed as a new biomarker to distinguish between multiple myeloma and lung adenocarcinoma (27). It has been found that DARS2 is an oncogene in hepatocellular carcinoma and can promote the progression of the hepatocellular carcinoma cell cycle while inhibiting apoptosis in HCC cells (16, 17). Although Wu’s team discovered that DARS2 can predict overall survival in BLCA and serve as a predictive model for assessing clinical outcomes (28). However, there have been no reported studies on the biological role of DARS2 in BLCA.

Our research findings indicate that patients with high DARS2 expression have a poorer prognosis. In terms of biological function, interfering with DARS2 expression can inhibit the proliferation, invasion, and migration of BLCA, while overexpression of DARS2 has been demonstrated to promote the malignant progression of tumors.

Our investigation into PD-L1 reveals that both the downregulation and upregulation of DARS2 may result in a corresponding decrease or increase in PD-L1 expression. Recent research has unveiled that knocking down DARS2 leads to a diminished expression of p-ERK1/2, while DARS2 overexpression prompts an elevation in p-ERK1/2 levels (29). Previous studies have substantiated the role of the ERK-MAPK pathway in modulating PD-L1 expression across diverse cell types, where the regulation of p-ERK1/2 expression can intricately impact PD-L1 levels (30–34). Drawing from these insights, we posit that DARS2 likely modulates PD-L1 expression through the regulation of p-ERK1/2.

In addition, our immune infiltration analysis indicates a positive correlation between the expression of DARS2 and CD8+ T cells. In the context of immunotherapy, CD8+ T cells play a pivotal role, secreting cytokines such as IFN-γ, TNF-α, and IL-2 to exert cytotoxic effects on tumor cells (35). In studies of bladder cancer prognostic markers, Wang found that CLIC1, as a prognostic marker, showed a negative correlation with CD8+ T cells. Patients with low CLIC1 expression exhibited increased infiltration of CD8+ T cells, correlating with a better prognosis (36). However, our study revealed that in patients with high DARS2 expression, there is an increased infiltration of CD8+ T cells, but paradoxically, a worse prognosis. Further classification of CD8+ T cells identified precursor-exhausted and terminally exhausted subtypes (37, 38). Precursor-exhausted CD8+ T cells typically lack effector molecules for tumor cell cytotoxicity, while terminally exhausted cells release cytokines for tumor killing but express high levels of immune checkpoints such as PD-1 (39). In contrast to Wang’s findings (36), we speculate that the upregulation of PD-L1 induced by high DARS2 expression may weaken the cytotoxicity of CD8+ T cells against tumor cells. Our experiments, involving DARS2 knockdown and overexpression, confirm concurrent upregulation or downregulation of PD-L1 with DARS2. Additionally, DARS2 knockdown led to a smaller decrease in IL-2 secretion by Jurkat cells in the co-culture system compared to the control group. IL-2 is a critical factor for T cell proliferation, survival, and immune function, while the binding of PD-L1/PD-1 induces CD8+ T cell apoptosis and loss of anti-tumor function (40–42). Therefore, highly infiltrated CD8+ T cells may not fully exert their cytotoxic effects in tumors due to the high expression of PD-L1 on the surface of tumor cells. Moreover, we observed that tumor cells with high DARS2 expression exhibit enhanced relative activity, while the knockdown group showed the opposite, further confirming the stronger immunosuppressive effect of tumor cells with high DARS2 expression.

On the other hand, we found a positive correlation between DARS2 expression and macrophage infiltration. Tumor-associated macrophages (TAMs) are immune cells in the tumor microenvironment expressing various immune checkpoints influencing immune function. TAMs can be classified into M1 and M2 types, with M1 playing a crucial role in innate immunity, while M2 is considered to have a protective effect on tumors (43). Multiple studies suggest that PD-L1 induces the conversion of M1 macrophages to the M2 phenotype, promoting tumor occurrence and development (44–47). The correlation between high DARS2 expression and PD-L1 may induce the conversion of macrophages to the M2 type, thereby weakening the immune cell’s cytotoxicity against tumors. In summary, high DARS2 expression may promote tumor development by altering the immune microenvironment of BLCA. Given DARS2’s impact on PD-L1 expression, it could potentially serve as a novel predictive indicator for immune therapy responses.

This study yielded several key findings. Firstly, we confirmed and validated the association of upregulated DARS2 expression with poor prognosis in BLCA. Secondly, we delved into the impact of DARS2 on the biological functions of bladder cancer cells, establishing a connection between DARS2 expression and an immunosuppressive tumor microenvironment, including its correlation with PD-L1 expression. This was further corroborated by our co-culture system, demonstrating the influence of DARS2 on the cytotoxicity of immune cells. Lastly, we conducted *in vivo* experiments to validate the impact of DARS2 knockdown on tumor formation. However, our study has some limitations. In one part, the sample size is relatively small, and the correlation between DARS2 and PD-L1 expression needs further validation in larger clinical cohorts. Another part, how DARS2 regulates the relationship between tumors and the immune microenvironment is not yet clear. In-depth investigations into these mechanisms may pave the way for novel immune therapy strategies, such as combining DARS2 inhibitors with immune checkpoint inhibitors to improve the prognosis of BLCA patients.

## Data availability statement

The original contributions presented in the study are included in the article/**Supplementary Material**. Further inquiries can be directed to the corresponding authors.

## Ethics statement

The studies involving humans were approved by the First Affiliated Hospital of Nanchang University. The studies were conducted in accordance with the local legislation and institutional requirements. The participants provided their written informed consent to participate in this study. The animal study was approved by the First Affiliated Hospital of Nanchang University. The study was conducted in accordance with the local legislation and institutional requirements.

## Author contributions

YH: Writing – original draft, Writing – review & editing. LM: Writing – review & editing. NJ: Writing – review & editing. WD: Writing – review & editing. XL: Writing – review & editing. BF: Writing – review & editing.
