# Supplementary figures and images for: Prognostic biomarker DARS2 correlated with immune infiltrates in bladder tumor
Source: Front Immunol. 2024 Jan 17;14:1301945. doi: 10.3389/fimmu.2023.1301945 (PMC10827901; doi:10.3389/fimmu.2023.1301945)

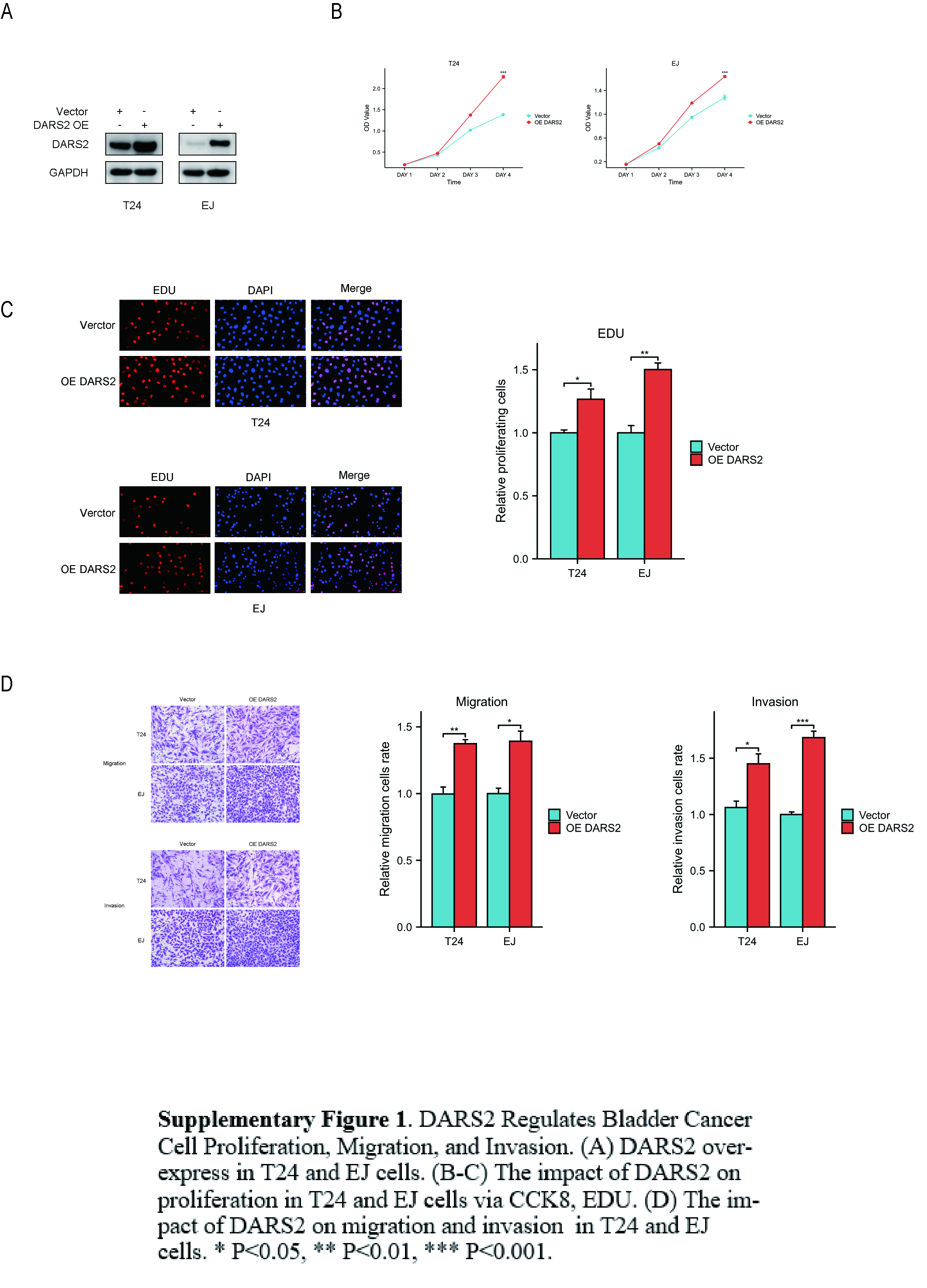

Supplement: Supplementary file 1 [file Image_1.tif]

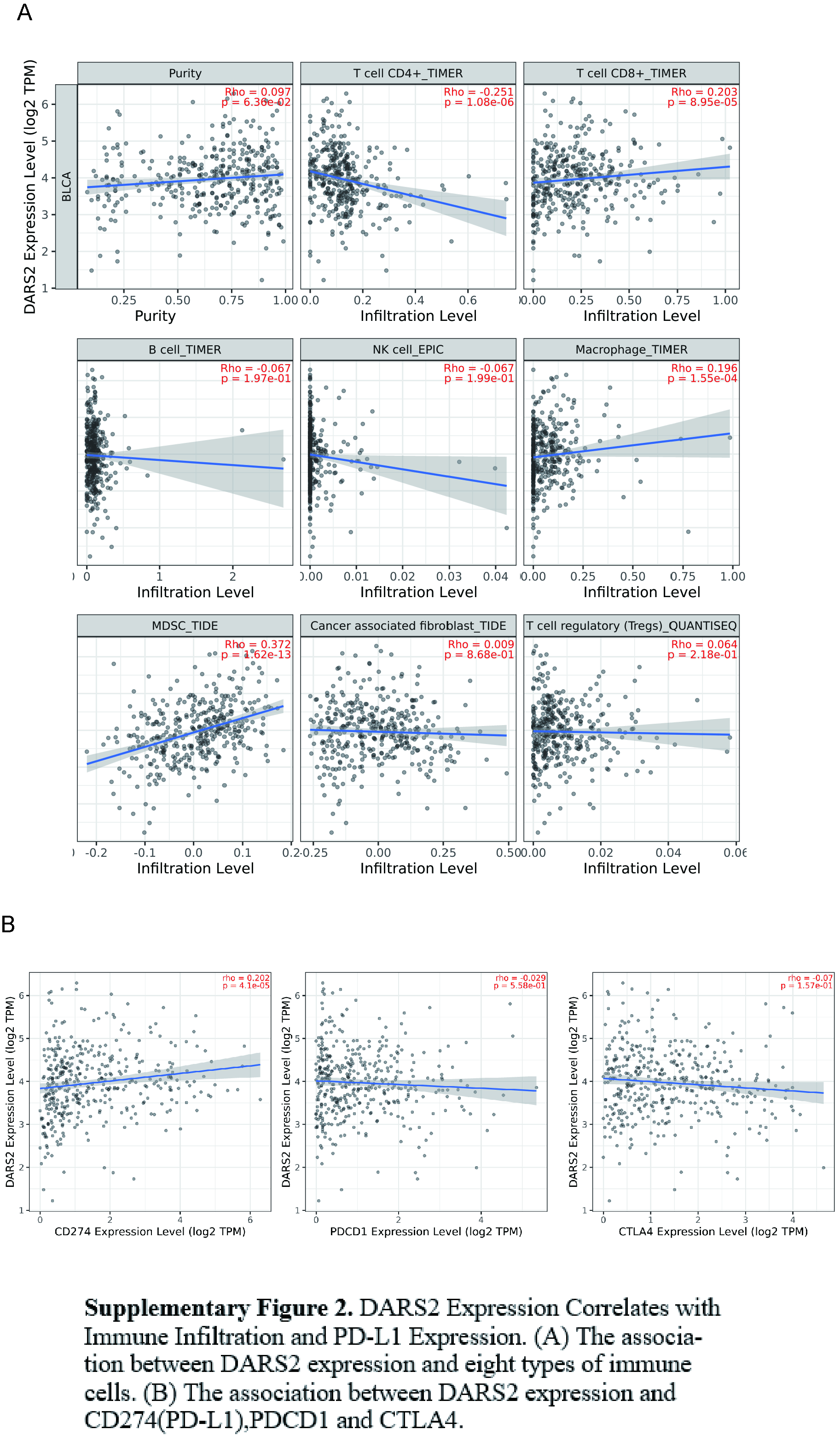

Supplement: Supplementary file 2 [file Image_2.tif]
