# Supplementary material for: Prognostic biomarker DARS2 correlated with immune infiltrates in bladder tumor
Source: Front Immunol. 2024 Jan 17;14:1301945. doi: 10.3389/fimmu.2023.1301945 (PMC10827901; doi:10.3389/fimmu.2023.1301945)
Supplement: Supplementary file 3 [file Table_1.docx]

**Supplement Table 1**

| Characteristics | Low expression of DARS2 | High expression of DARS2 | P value |
| --- | --- | --- | --- |
| n | 206 | 206 |  |
| Pathologic T stage, n (%) |  |  | 0.036 |
| T1&T2 | 73 (19.3%) | 50 (13.2%) |  |
| T3 | 91 (24.1%) | 105 (27.8%) |  |
| T4 | 25 (6.6%) | 34 (9%) |  |
| Pathologic N stage, n (%) |  |  | 0.064 |
| N0 | 129 (35.1%) | 109 (29.6%) |  |
| N1 | 20 (5.4%) | 26 (7.1%) |  |
| N2&N3 | 34 (9.2%) | 50 (13.6%) |  |
| Pathologic M stage, n (%) |  |  | 0.007 |
| M0 | 102 (48.1%) | 99 (46.7%) |  |
| M1 | 1 (0.5%) | 10 (4.7%) |  |
| Pathologic stage, n (%) |  |  | 0.014 |
| Stage I | 2 (0.5%) | 2 (0.5%) |  |
| Stage II | 79 (19.3%) | 50 (12.2%) |  |
| Stage III | 68 (16.6%) | 74 (18%) |  |
| Stage IV | 56 (13.7%) | 79 (19.3%) |  |
| Gender, n (%) |  |  | 0.823 |
| Female | 55 (13.3%) | 53 (12.9%) |  |
| Male | 151 (36.7%) | 153 (37.1%) |  |
| Age, n (%) |  |  | 0.551 |
| <= 70 | 113 (27.4%) | 119 (28.9%) |  |
| > 70 | 93 (22.6%) | 87 (21.1%) |  |
| BMI, n (%) |  |  | 0.645 |
| <= 25 | 79 (21.8%) | 73 (20.2%) |  |
| > 25 | 104 (28.7%) | 106 (29.3%) |  |
| Histologic grade, n (%) |  |  | < 0.001 |
| High grade | 186 (45.5%) | 202 (49.4%) |  |
| Low grade | 19 (4.6%) | 2 (0.5%) |  |
| Smoker, n (%) |  |  | 0.253 |
| No | 60 (15%) | 49 (12.3%) |  |
| Yes | 141 (35.3%) | 149 (37.3%) |  |

**Supplement Table 2**

| Characteristics | Low expression of DARS2 | High expression of DARS2 | P value |
| --- | --- | --- | --- |
| n | 19 | 18 |  |
| Pathologic T stage, n (%) |  |  | 0.103 |
| T3 & T4 | 7 (36.8%) | 12 (66.7%) |  |
| T1 & T2 | 12 (63.2%) | 6 (33.3%) |  |
| Pathologic N stage, n (%) |  |  | 0.042 |
| N1 & N2 | 1 (5.3%) | 6 (33.3%) |  |
| N0 | 18 (94.7%) | 12 (66.7%) |  |
| Pathologic M stage, n (%) |  |  | 0.042 |
| M1 & M2 | 1 (5.3%) | 6 (33.3%) |  |
| M0 | 18 (94.7%) | 12 (66.7%) |  |
| Histologic grade, n (%) |  |  | 0.046 |
| High grade | 14 (73.7%) | 18 (100%) |  |
| Low grade | 5 (26.3%) | 0 (0%) |  |
| Gender, n (%) |  |  | 0.230 |
| 1 | 19 (100%) | 16 (88.9%) |  |
| 2 | 0 (0%) | 2 (11.1%) |  |
| Age, n (%) |  |  | 1.000 |
| <=70 | 12 (63.2%) | 11 (61.1%) |  |
| >70 | 7 (36.8%) | 7 (38.9%) |  |

**Supplement Table 3**

| Characteristics | Total(N) | Univariate analysis | |  | Multivariate analysis | |
| --- | --- | --- | --- | --- | --- | --- |
|  |  | Hazard ratio (95% CI) | P value |  | Hazard ratio (95% CI) | P value |
| Pathologic T stage | 377 |  |  |  |  |  |
| T1&T2 | 123 | Reference |  |  | Reference |  |
| T3 | 195 | 1.970 (1.339 - 2.899) | **< 0.001** |  | 1.698 (0.880 - 3.277) | 0.114 |
| T4 | 59 | 2.987 (1.860 - 4.797) | **< 0.001** |  | 1.986 (0.902 - 4.374) | 0.089 |
| Pathologic N stage | 367 |  |  |  |  |  |
| N0 | 238 | Reference |  |  | Reference |  |
| N2&N3&N1 | 129 | 2.250 (1.649 - 3.072) | **< 0.001** |  | 1.745 (1.057 - 2.880) | **0.029** |
| Pathologic M stage | 212 |  |  |  |  |  |
| M0 | 201 | Reference |  |  | Reference |  |
| M1 | 11 | 3.112 (1.491 - 6.493) | **0.002** |  | 1.202 (0.460 - 3.141) | 0.707 |
| Age | 411 |  |  |  |  |  |
| <= 70 | 231 | Reference |  |  | Reference |  |
| > 70 | 180 | 1.424 (1.064 - 1.906) | **0.018** |  | 1.351 (0.835 - 2.187) | 0.221 |
| Gender | 411 |  |  |  |  |  |
| Male | 303 | Reference |  |  |  |  |
| Female | 108 | 1.152 (0.835 - 1.590) | 0.390 |  |  |  |
| Histologic grade | 408 |  |  |  |  |  |
| Low grade | 21 | Reference |  |  | Reference |  |
| High grade | 387 | 2.960 (0.732 - 11.959) | 0.128 |  | 1.464 (0.187 - 11.468) | 0.717 |
| DARS2 | 411 |  |  |  |  |  |
| Low | 206 | Reference |  |  | Reference |  |
| High | 205 | 1.480 (1.102 - 1.987) | **0.009** |  | 1.953 (1.167 - 3.270) | **0.011** |
